# Outcomes of patients enrolled in an antiretroviral adherence club with recent viral suppression after experiencing elevated viral loads

**DOI:** 10.4102/sajhivmed.v20i1.905

**Published:** 2019-06-11

**Authors:** Joseph Sharp, Lynne Wilkinson, Vivian Cox, Carol Cragg, Gilles van Cutsem, Anna Grimsrud

**Affiliations:** 1 Emory University School of Medicine, Atlanta, United States; 2 Médecins Sans Frontières, Cape Town, South Africa; 3 Centre for Infectious Disease Epidemiology and Research, School of Public Health and Family Medicine, University of Cape Town, Cape Town, South Africa; 4 International AIDS Society, Cape Town, South Africa; 5 Provincial Department of Health, Western Cape, Cape Town, South Africa

**Keywords:** Differentiated care, Retention, Viral suppression, Adherence, High-risk patients, ART delivery

## Abstract

**Background:**

Eligibility for differentiated antiretroviral therapy (ART) delivery models has to date been limited to low-risk stable patients.

**Objectives:**

We examined the outcomes of patients who accessed their care and treatment through an ART adherence club (AC), a differentiated ART delivery model, immediately following receiving support to achieve viral suppression after experiencing elevated viral loads (VLs) at a high-burden ART clinic in Khayelitsha, South Africa.

**Methods:**

Beginning in February 2012, patients with VLs above 400 copies/mL either on first- or second-line regimens received a structured intervention developed for patients at risk of treatment failure. Patients who successfully suppressed either on the same regimen or after regimen switch were offered immediate enrolment in an AC facilitated by a lay community health worker. We conducted a retrospective cohort analysis of patients who enrolled in an AC directly after receiving suppression support. We analysed outcomes (retention in care, retention in AC care and viral rebound) using Kaplan–Meier methods with follow-up from October 2012 to June 2015.

**Results:**

A total of 165 patients were enrolled in an AC following suppression (81.8% female, median age 36.2 years). At the closure of the study, 119 patients (72.0%) were virally suppressed and 148 patients (89.0%) were retained in care. Six, 12 and 18 months after AC enrolment, retention in care was estimated at 98.0%, 95.0% and 89.0%, respectively. Viral suppression was estimated to be maintained by 90.0%, 84.0% and 75.0% of patients at 6, 12 and 18 months after AC enrolment, respectively.

**Conclusion:**

Our findings suggest that patients who struggled to achieve or maintain viral suppression in routine clinic care can have good retention and viral suppression outcomes in ACs, a differentiated ART delivery model, following suppression support.

## Introduction

The introduction of antiretroviral therapy (ART) for the treatment of HIV has led to massive reductions in mortality and slowed the progression of disease and transmission of infection.^1,2^ These reductions are contingent upon strict adherence to ART regimens and long-term retention in care.^3,4^ Treatment programmes throughout the world are both expanding to meet the Joint United Nations Programme on HIV/AIDS (UNAIDS) 90-90-90 targets and continuing to mature, as the first patients initiated in some treatment programmes will soon enter their third decade on ART.^5^ Despite advances in the reduction of the costs of treatment,^6^ the stigma associated with infection^7^ and the need to integrate treatment into daily routines,^8,9^ increasing numbers of patients are interrupting care and experiencing viral rebound. While retention has been consistent between 74% and 78% at 12 months from 2005 to 2013,^10^ the number of patients in care globally has increased dramatically from 1.3 million in 2005^11^ to over 21.7 million in 2017.^12^ This trend is evident in South Africa where the treatment programme has grown from just less than 100 000 to over 4 million patients, and concurrently, the number of patients interrupting or abandoning care has also increased.^13^ Models of care must adapt to focus on the needs of the growing population that has interrupted ART while supporting quality care for the new patients eligible for ART as countries adopt ‘test and start’ guidelines.

Differentiated ART delivery models such as ART adherence clubs (ACs) have been shown to be successful and cost-effective in providing treatment, care and support.^14,15,16,17^ These models have traditionally been restricted to clinically stable patients, defined as patients on ART for 12 months or more with two undetectable viral loads (VLs). Differentiated ART delivery models promote adherence by reducing the frequency of visits and time spent in a clinic, allowing for increased peer and lay healthcare worker (LHCW) support and ensuring longer ART supply.^18,19,20,21,22,23^ If such models of ART delivery remain restricted to low-risk stable patients on first-line treatment, the growing cohort of patients struggling with adherence may be left behind, stuck in delivery models that already failed them. Furthermore, it may be the patients who are not stable, those at risk of treatment failure, who stand to gain the most from simplifying their ART refill delivery mechanism.^24^ While differentiated ART delivery models have received widespread attention and have been incorporated into the World Health Organization’s treatment guidelines,^25^ they have been restricted to low-risk stable patients. Data on the outcomes of patients at high-risk of experiencing viral rebound who access differentiated ART delivery models do not currently exist. We describe the outcomes of patients referred directly to ACs after viral suppression following specific adherence support.

## Research methods and design

### Study design

A descriptive retrospective cohort study was undertaken using routine data collected under programmatic conditions at Ubuntu Clinic, Khayelitsha, Western Cape, South Africa, for patients who joined ACs between February 2012 and February 2014 after viral suppression following the risk of treatment failure (ROTF) intervention.

### Setting

The study was conducted at Ubuntu Clinic in Khayelitsha, South Africa. Khayelitsha is a township in Cape Town with a population of approximately half a million people and high rates of HIV and tuberculosis (TB). In 2011, the antenatal HIV prevalence was 34%.^26,27^ The community is largely poor, with 55% of the population living in informal housing and 60% unemployment among working age individuals.^28^ In 2001, Ubuntu Clinic became the first public sector clinic in the country to provide ART;^29^ by March 2017, 10 252 patients were retained in ART care at Ubuntu Clinic, with close to 40 000 patients in ART care in Khayelitsha sub-district (a sub-district in the Cape Metro district).

### Adherence club model and risk of treatment failure intervention

The AC model has been described in detail previously.^15,16,17,18^ Briefly, in the Western Cape, clients were initially regarded as stable and eligible for the AC model if on ART for 12 months or more with two undetectable VLs and no co-morbidities requiring frequent clinical assessment. In 2015, stability criteria changed to on ART for 6 months or more with a single undetectable VL. Adherence clubs were composed of approximately 30 patients who met with an LHCW five times a year (every 2 months except over year-end holidays when a 4-month ART refill was provided) for a short symptom screen, peer support and distribution of pre-packed ART refills. Some ACs were facility based and met at the Ubuntu Clinic, while others were decentralised to community venues. Adherence club patients had an annual blood draw and an annual clinical consultation as part of their AC visit schedule. If a patient experienced viral rebound (VL > 400) in the AC, failed to attend their AC or became clinically unstable for any reason, the patient was referred back into routine clinic care for ongoing management. The AC model was brought to scale in the Cape Town health district with 40.9% (62 874 patients) of all ART patients in the district accessing ART care and support through ACs by the end of 2016.^30^ Twenty-four-month retention, annual VL completion and viral suppression outcomes^31^ were 89.3%, 88.1% and 97.2%, respectively.

In 2012, the ROTF intervention was piloted at Ubuntu Clinic by Médecins Sans Frontières (MSF) and the Western Cape Department of Health.^32,33,^ The Western Cape Department of Health has subsequently adopted the intervention to manage all patients failing or at risk of failing ART with phased implementation in all its Cape Town facilities starting at the end of 2015. The intervention was designed to provide integrated adherence support and clinical management for all patients in routine clinical care with VLs above 400 copies/mL, irrespective of treatment regimen. Patients who experienced a single VL > 400 copies/mL were enrolled in an LHCW support group, while those with two consecutive VLs > 400 copies/mL experienced more intensive counselling with a nurse trained to provide integrated adherence and clinical management. Adherence was managed through structured steps including VL monitoring and switching patients to second-line ART regimens in accordance with national guidelines (two consecutive VLs > 1000 copies/mL).

After suppression (VL < 400 copies/mL) – whether on first line, after switch to second line or on second line – patients were given the choice to enrol directly into an AC or return to routine clinician-led facility-based care. Patients who suppressed and enrolled in an AC following the ROTF intervention are hereafter referred to as ‘high-risk patients’ as they were regarded to be at a higher risk of interrupting their treatment again.^34,35^ High-risk patients were enrolled in ACs on a rolling basis, and therefore ACs are composed of both stable and high-risk patients.

### Data collection and analysis

#### Analysis inclusion and exclusion

**FIGURE 1:**
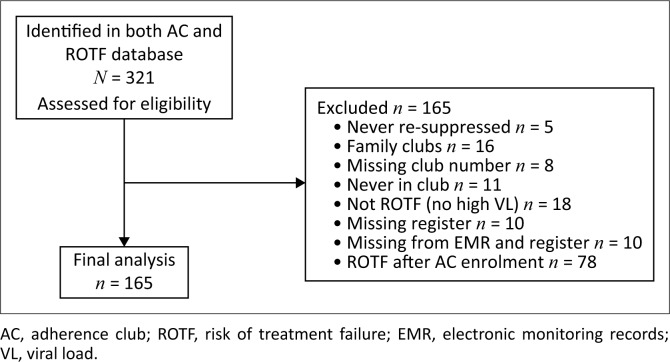
Flow chart of analysis inclusion criteria applied to obtain study sample.

Patients who joined ACs between February 2012 and February 2014 after suppressing in the ROTF intervention were identified retrospectively by comparing the clinic’s electronic monitoring records (EMR), which identified patients participating in the ROTF and AC programmes. Additional data were gathered from AC registers on those patients who were identified as having participated in both programmes. Patients were excluded if they were enrolled in an AC *before* the ROTF intervention, enrolled in a family AC (utilised for children and their caregivers), missing from the AC register or confirmed to have never joined an AC (indicating EMR AC participation incorrect), never suppressed after ROTF or if they never had a VL greater than 400 copies/mL (indicating EMR ROTF participation incorrect) (Figure 1). One AC register could not be found, and all patients referred to that AC were excluded from analysis. This left only high-risk patients confirmed to have joined ACs directly after participation and successful suppression following the ROTF intervention.

#### Data collection

Data for each patient in the analysis cohort were collected from patient visit and laboratory data from the EMR and AC registers. Missing VL results were obtained from the National Health Laboratory Service database. Patient clinic folders were consulted for patients whose most recent status was missing from the AC registers to confirm their current AC status. Key variables collected included ART regimen, ART start date, ROTF enrolment date, last unsuppressed VL and date, first suppressed VL and date, all VLs and dates after club enrolment and all clinic and club visits after suppression.

#### Statistical analysis

Patients entered the analysis on their first AC date (between February 2012 and February 2014) and were followed until March 24, 2015. We assessed three outcomes: retention in care, retention in club care and viral suppression. Retention in care was defined as having contact with the clinic or AC between March 24 and June 21, 2015, with retention in club care defined as attending an AC in the same period. Patients were classified as virally suppressed if their *last VL before analysis closure* was less than 400 copies/mL. We define viral rebound as an elevated VL above 400 copies/mL after having achieved viral suppression. Known deaths and transfers contributed retention time until they were censored at the time of death or transfer.

Patient characteristics at enrolment into an AC (gender, age at ART start, age at AC start, year of ART start, treatment regimen) and time from ART initiation to ROTF participation and from ROTF participation to AC enrolment were summarised using medians and interquartile ranges (IQRs) for continuous variables and proportions for categorical variables. Cross-sectional retention outcomes are reported at study closure. Kaplan–Meier methods were used to estimate the survival probabilities of retention, AC retention and viral suppression, and are reported at 3-monthly intervals to 18 months with 95% confidence intervals (CIs).

Data were analysed using Stata 13.0 software (STATA Corporation, College Station, TX, US).

### Ethical consideration

Because of the nature of the study, individual patient consent was not obtained, consistent with the Declaration of Helsinki. All participants and data were drawn from an ongoing cohort study of routine ART outcomes in Khayelitsha, Cape Town, approved by the Human Research Ethics Committee of the Faculty of Health Sciences at the University of Cape Town (HREC 395/2005). Only routine clinical service data were used and no identifying patient information was entered into the database.

## Results

### Patient characteristics

From February 2012 to February 2014, 165 high-risk patients who completed the ROTF intervention and suppressed were immediately enrolled in an AC. The cohort was predominantly female (81.8%) with a median age at ART start of 31 years (IQR: 28–37). Current treatment regimens were available for 133 patients, and of those 105 (79%) were on second-line therapy (Table 1) at the time of AC enrolment. The median time from ART initiation to enrolment in the ROTF intervention was 3.4 years (IQR: 2.1–5.5), and the median time from ROTF intervention to AC enrolment was 1.2 years (IQR: 1.0–1.5).

**TABLE 1:** Description of risk of treatment failure patients who suppressed and were referred to an adherence club.

Characteristic	*N* = 165			
	*N*	%	Median	IQR
**Gender**				
Males	30	18.2	-	-
Females	135	81.8	-	-
**Age ART start**	-	-	30.7	27.6–37.1
**Age**				
16–19	3	1.8	-	-
20–24	16	9.7	-	-
25–34	89	54.9	-	-
35–44	45	27.3	-	-
45+	12	7.3	-	-
**Age ART start**	-	-	36.2	32.2–32.4
**Categorical**	
16–19	0	0.0	-	-
20–24	1	0.6	-	-
25–34	66	40.0	-	-
35–44	69	41.8	-	-
45+	29	17.6	-	-
**Year of ART start**	
2002–2005	27	16.4	-	-
2006–2008	56	33.9	-	-
2009–2010	51	30.9	-	-
2011–2013	31	18.8	-	-
**Regimen at AC start**	
First line	28	21.1	-	
Second line	105	79.0	-	
**Median time from ART start to ROTF, years**	**-**	**-**	**3.4**	**2.1–5.5**
**Median time from ART start to AC start, years**	**-**	**-**	**4.7**	**3.4–7.2**
**Median time from ROTF start to AC start, years**	**-**	**-**	**1.2**	**1.0–1.5**

ART, antiretroviral therapy; IQR, interquartile range; AC, adherence club; ROTF, risk of treatment failure.

### Cross-sectional outcomes

During the study period, two patients (1.2%) died, 15 (7.8%) were lost to follow-up and 40 (24.0%) experienced viral rebound. At the closure of the study, 148 patients (89.0%) were retained in care and 119 patients (72.0%) were virally suppressed. When stratified by known ART regimen, 26 patients (93.0%) on first line and 97 patients (92.0%) on second line were retained in care, while 20 patients (71.0%) on first line and 83 patients (79.0%) on second line were virally suppressed.

### Time to event outcomes

**FIGURE 2:**
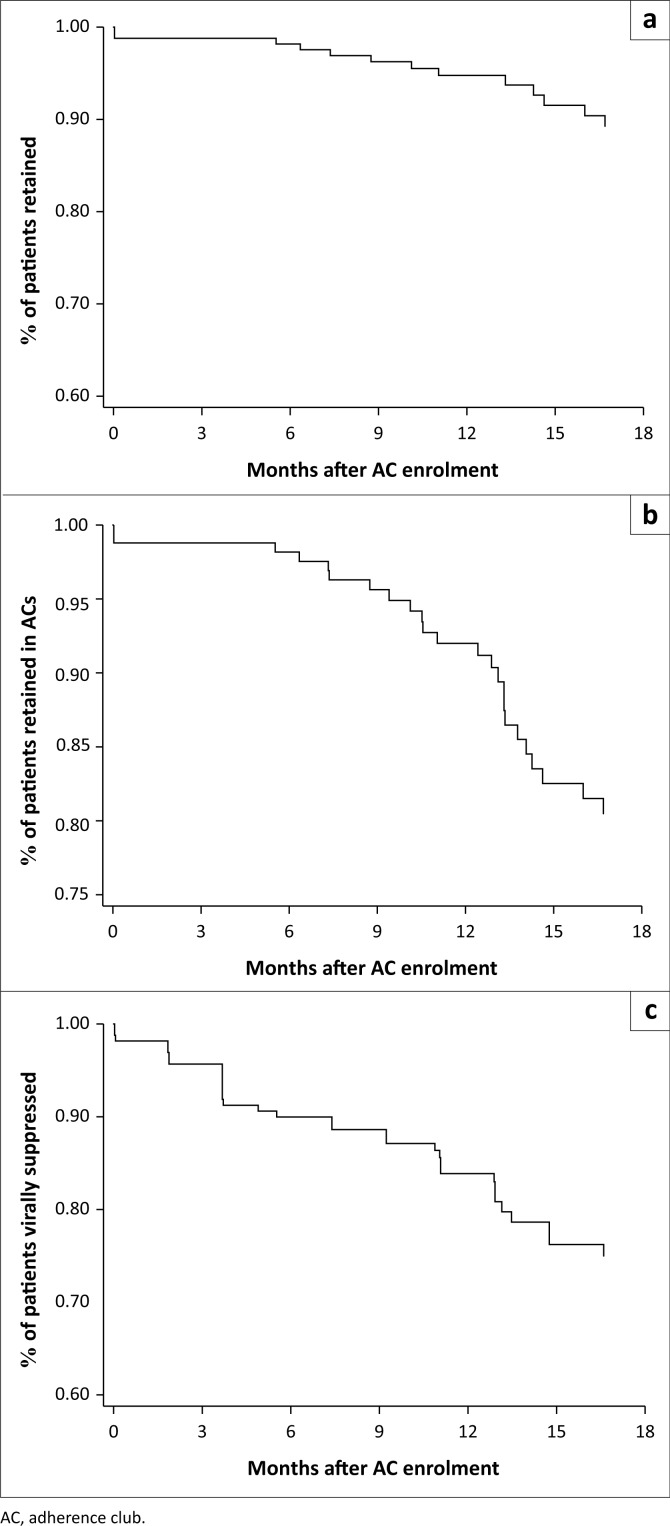
Retention in care (a), retention in adherence club care (b) and viral suppression (c) over the first 18 months in adherence clubs immediately after viral suppression and referral to an adherence club.

Retention in care was estimated to be 98.8% (95% CI, 94.4–99.4), 94.8% (95% CI, 89.8–97.4) and 89.3% (95% CI, 81.8–93.8) at 6, 12 and 18 months after AC enrolment, respectively (Table 2, Figure 2a). Retention in AC care was estimated to be 98.2% (95% CI, 94.4–99.4), 92.0% (95% CI, 86.3–95.4) and 80.5% (95% CI, 72.0–86.6) over the same time periods (Table 2, Figure 2b). Eighteen months after enrolment in ACs, 90% of patients retained in clinic care were still in ACs. Viral suppression was estimated to be 90.0% (95% CI, 84.1–93.7), 83.9% (95% CI, 76.8–88.9) and 75.0% (95% CI, 66.0–81.9) at 6, 12 and 18 months after AC enrolment, respectively (Table 2, Figure 2c).

**TABLE 2:** Kaplan–Meier estimates of retention in care, retention in adherence club care and viral suppression by duration of follow-up after first AC meeting.

Duration of follow-up	*n*	%	Retention in care			Retention in AC care			Viral suppression		
			Events	%	95% CI	Events	%	95% CI	Events	%	95% CI
3 months	160	97.0	2	98.8	95.2–99.7	2	98.8	95.2–99.7	7	95.7	91.1–97.9
6 months	159	96.3	1	98.2	94.4–99.4	1	98.2	94.4–99.4	9	90.0	84.1–93.7
9 months	145	87.9	3	96.2	91.8–98.3	4	95.6	91.0–97.9	2	88.6	82.5–92.7
12 months	127	77.0	2	94.8	89.8–97.4	5	92.0	86.3–95.4	6	83.9	76.8–88.9
15 months	82	49.7	3	91.5	84.9–95.3	10	82.5	74.4–88.3	7	76.2	67.4–82.9
18 months	80	48.5	2	89.3	81.8–93.8	2	80.5	72.0–86.6	1	75.0	66.0–81.9

AC, adherence club; CI, confidence interval.

## Discussion

Patients who had previously had elevated VLs had good treatment outcomes following supported viral suppression and direct referral to ACs, a differentiated ART delivery model. Despite having had recent elevated VL, 75% of patients who joined ACs after undergoing a ROTF intervention were estimated to maintain viral suppression 18 months after joining the AC. Eighteen months after AC enrolment, retention in care was estimated at 89%, and 90% of patients retained in care were still in ACs. Care was differentiated in the intensified management intervention to target patients failing treatment and in the ART delivery model provided immediately after suppression. To date there is limited evidence on the outcomes of high-risk patients in ART delivery models differentiated for stable patients.

We observed retention and viral suppression outcomes matching or exceeding those of retention benchmarks and meta-analyses from sub-Saharan Africa through 18 months of follow-up. Retention in sub-Saharan Africa was estimated to be 81% at 12 months,^10^ significantly less than the 94.8% retention in this cohort of recently suppressed patients. While we report 12-month retention from AC enrolment, not ART initiation, this remains significant. Twelve-month retention is only slightly lower than the 12-month 97.0% retention observed in stable patients in ACs at the same clinic^15^ and the 99.0% retention observed in stable patients in a similar community AC cohort^36^ and the 12-month retention in ACs across the Cape Metro.^31^ In a 2015 systematic review of VL suppression, 12-month suppression in sub-Saharan Africa was estimated to be 64.2%.^37^ We observed 83.9% viral suppression at 12 months after AC enrolment. These outcomes also compare well to those of patients switched to second-line regimens. Analysis of a cohort in Durban, South Africa, found 25.0% virological failure every 6 months after switching to second-line regimens.^38^ In a European cohort of treatment-experienced patients who recently achieved viral suppression, 31.0% of patients experienced viral rebound within 1 year.^35^ Considering 100.0% of the study cohort experienced recent elevated VLs and 79.0% had either recently been switched to a second-line regimen within the ROTF intervention or entered ROTF on a second-line regimen, our results are promising.

Ninety per cent of patients retained in care after 18 months were still receiving their care in ACs, suggesting a high level of satisfaction with the service delivery model. This result is important because patient satisfaction is a strong predictor of adherence to ART regimens.^39^ While patients on ART experience elevated VLs (including viral rebound) for a variety of reasons, the single largest predictor is sub-optimal treatment adherence.^4,40,41,42,43^ Therefore, it follows that patients have better outcomes in models that better fit their lives. The AC model may support patients struggling with adherence in routine care by reducing or removing barriers to adherence.

In addition to the differentiated nature of the ACs, the model was also differentiated from routine care, providing more intensified integrated adherence and clinical care through the ROTF intervention to meet the needs of patients as they attempted to achieve viral suppression after elevated VLs and remain in care. This model of VL-informed differentiated care has been shown to be effective and cost-efficient in supporting patients who experience elevated VLs in routine care.^44^ In other words, it is possible that both the intense support in achieving suppression through the ROTF and simplifying ongoing access to care and treatment, with peer support, through the AC model immediately after suppression could be responsible for the positive outcomes.

These results should be viewed in light of a number of limitations. Firstly, a control group was not obtained, making comparison of these results difficult. Because of the retrospective nature of the study and the ability of patients to self-select into AC care or routine clinic care after ROTF, any control group chosen would be biased. We chose to compare the outcomes to broader benchmark goals for all ART programmes. Importantly, our analysis begins at AC enrolment and not ART start. Because the largest drop in retention occurs immediately after ART start, care must be taken when comparing these results to those of newly enrolled patients. Secondly, tracing of patients lost to follow-up to identify undocumented transfers was not completed. However, any bias this limitation created would serve to reduce observed retention. Thirdly, patients were given the choice to join an AC or return to routine clinic care after completing the ROTF intervention. It is therefore possible that only the motivated patients joined ACs, and our results are not representative of all patients who have experienced an elevated VL. This scenario is unlikely because fewer than 10.0% of patients chose to return to routine care after achieving suppression. Regardless, by allowing patients to self-select into care models, the probability that they will find a model of care that suits their life, and thus maintain adherence, increases. Fourthly, it is possible that transmitted resistance to first-line regimens was responsible for the positive response in patients switched to second line. This would only account for a small proportion of patients given the relative infrequency of transmitted resistance in South Africa^45^ and the extensive evidence indicating that non-adherence is the primary cause of an elevated VL.^32,46^ It is also possible that patients switched to second-line therapy had positive viral suppression results despite continued poor adherence because of a switching effect.^47^ However, this effect is thought to be minimal as most patients who fail second-line treatment after switching do so within the first 2 years and the outcome would be seen within our follow-up period.^48^ Unfortunately, we are unable to differentiate the patients who entered the ROTF intervention on second-line regimens from those who were switched to second line during the intervention and are susceptible to this switching effect. In addition, because of the limited number of patients, there was insufficient power to analyse associations between outcomes and patient demographics. An attempt was made to include all patients who participated in both ROTF and ACs by cross-referencing both databases; however, it is possible that patients were missed in our sampling approach. In addition, data were collected from routine clinical databases and thus may be subject to data quality error. Finally, it is possible that the exclusion criteria that were applied biased the results towards increased retention and viral suppression. This possibility was minimised by cross-checking multiple data sources and excluding the entire AC when information was missing for a selective subset of patients in that AC. Despite these limitations, we find these results promising in introducing the idea that patients who experienced a recent elevated VL can have positive outcomes if care is differentiated to meet their specific needs at a particular time point in their treatment pathway.

## Conclusion

Further research is needed to fully understand how less intense, differentiated ART delivery models can collectively support the heterogeneous population of patients currently ineligible for these models. We recommend both small-scale implementation in diverse contexts to assess the generalisability of our findings and randomised control trials to directly compare the outcomes of patients experiencing elevated VLs recently suppressed or resuppressed immediately accessing a simplified ART delivery model versus routine care. Tests of association should be employed to determine the populations who could most benefit. Finally, retention in AC care had its sharpest decline between 12 and 18 months, and longer term follow-up is needed to determine if differentiated ART delivery models can support patient retention over the long run. In summary, criteria for differentiation must continue to be re-evaluated. Using the criteria of proven ‘stability’ may exclude those who have the most to gain from streamlining access to ART, including those who have recently suppressed.

In conclusion, our findings suggest that patients who struggled to achieve or maintain viral suppression in routine clinic care can have good retention and viral suppression outcomes in differentiated ART delivery models, such as ACs, immediately following suppression support. These models may remove health system barriers imposed by clinician-led facility-based models. Further studies are required to evaluate both retention and viral suppression benefits of expanding access to differentiated ART delivery models to patients who have struggled with adherence.
